# DCT-Based Preprocessing Approach for ICA in Hyperspectral Data Analysis

**DOI:** 10.3390/s18041138

**Published:** 2018-04-08

**Authors:** Kamel Boukhechba, Huayi Wu, Razika Bazine

**Affiliations:** The State Key Laboratory of Information Engineering in Surveying, Mapping, and Remote Sensing, Wuhan University, Wuhan 430079, China; wuhuayi@whu.edu.cn (H.W.); razika022002@yahoo.fr (R.B.)

**Keywords:** discrete cosine transform, hyperspectral dimensionality reduction, independent component analysis, hyperspectral signal subspace identification by the minimum error, principal component analysis

## Abstract

The huge quantity of information and the high spectral resolution of hyperspectral imagery present a challenge when performing traditional processing techniques such as classification. Dimensionality and noise reduction improves both efficiency and accuracy, while retaining essential information. Among the many dimensionality reduction methods, Independent Component Analysis (ICA) is one of the most popular techniques. However, ICA is computationally costly, and given the absence of specific criteria for component selection, constrains its application in high-dimension data analysis. To overcome this limitation, we propose a novel approach that applies Discrete Cosine Transform (DCT) as preprocessing for ICA. Our method exploits the unique capacity of DCT to pack signal energy in few low-frequency coefficients, thus reducing noise and computation time. Subsequently, ICA is applied on this reduced data to make the output components as independent as possible for subsequent hyperspectral classification. To evaluate this novel approach, the reduced data using (1) ICA without preprocessing; (2) ICA with the commonly used preprocessing techniques which is Principal Component Analysis (PCA); and (3) ICA with DCT preprocessing are tested with Support Vector Machine (SVM) and K-Nearest Neighbor (K-NN) classifiers on two real hyperspectral datasets. Experimental results in both instances indicate that data after our proposed DCT preprocessing method combined with ICA yields superior hyperspectral classification accuracy.

## 1. Introduction

Hyperspectral imagery contains hundreds of bands at a very high spectral resolution providing detailed information about objects, making hyperspectral imagery appropriate for source separation and classification. However, this high-dimensional data increases computation time and decreases the effectiveness of a classifier [1], which is one consequence of the curse of dimensionality [2]. Therefore, hyperspectral images require preprocessing to reduce spectral bands [3,4] and denoising prior to further processing. Many techniques have been applied in hyperspectral data analysis to reduce data dimensionality, including selection-based [5] and transformation-based techniques [6] such as Independent Component Analysis (ICA).

ICA is a popular unsupervised Blind Source Separation (BSS) technique [7], which determines statistically independent components. Unlike correlation-based transformations such as Principal Component Analysis (PCA), ICA not only decorrelates signals but also makes the signals as independent as possible [8]. ICA accurately recognizes patterns, reduces noise and data effectively, and is widely applied in systems involving multivariable data [9]. The general idea is to reduce high dimension space to a lower dimension with the transformed components describing the essential structure of the data; containing the relevant information, but without the volume of the original data.

ICA has been applied to hyperspectral data for dimensionality reduction, for source separation, and data compression [9]. It however, is computationally expensive and does not have specific criteria for selecting components [9], which limits its usefulness in high-dimensional data analysis. To overcome this limitation, PCA is the most commonly used preprocessing technique in ICA applications for hyperspectral data dimensionality reduction [10].

In this paper, the Discrete Cosine Transform (DCT) is proposed as a data preprocessing procedure for ICA to reduce dimension, noise, and computation time. DCT is among the filters based on orthogonal transforms that have demonstrated good performance in additive white Gaussian noise removal [11,12], which is the dominant noise component in hyperspectral imagery [13,14,15]. However, very few studies have taken advantage of DCT in hyperspectral imagery. For instance, spatial DCT was proposed as preprocessing for source separation exploiting the inter-pixel spatial correlation in [16].

By exploiting DCT ability to concentrate signal energy in few low-frequency coefficients, our procedure considers each pixel vector as a 1-D discrete signal to obtain its frequency domain profile. In the frequency domain, we can easily retain the most useful information as represented by these few low-frequency components [17], and discards high-frequency components that generally represent noise. Therefore, by performing DCT, we can either reduce data dimensionality and noise.

Therefore, our objective was to implement and test a new DCT-based preprocessing procedure for ICA that overcomes the limits of ICA regarding computational cost and make it more effective for dimensionality reduction in hyperspectral imagery analysis. To evaluate this procedure, SVM and K-NN classifiers were applied on the reduced data using ICA only, on ICA with PCA as preprocessing procedure and on ICA with our proposed DCT preprocessing procedure.

The rest of this paper is organized as follows. Section 2 briefly reviews the mathematical formulation of ICA and DCT. Section 3 describes the proposed approach. Section 4 presents datasets, experimental setup, evaluation process and illustrates the results. In Section 5, some conclusions are drawn.

## 2. ICA

The main idea behind ICA assumes that data are linearly mixed by a set of separate independent sources, and that it is possible to decompose these signal sources according to measurements of their statistical independence. ICA analysis is applied in spectral un-mixing, and anomaly and target detection [18]. There are several applications specifically designed for remote sensing imagery [19,20,21]. Indeed, the ICA transform is based on a non-Gaussian assumption of independence among sources, a usual characteristic of hyperspectral datasets [22]. To measure source independence, different criteria have been proposed [7], most are based on the concept of mutual information as a criterion to measure the discrepancy between two random sources [23].

Given an observation vector with *x* = [*x*_0_, *x*_1_, *x*_2_, …, *x_P_*_−1_] as a linear mixture of *P* independent elements of a random vector source *s* = [*s*_0_, *s*_1_, *s*_2_, …, *s_P_*_−1_]. In matrix terms, the model is given by [7]:

*X* = *A*·*S* (1)

where, *A* represents a mixing matrix.

ICA estimates an unmixing matrix *W* (i.e., the inverse of *A*) to give the best possible approximation of *S*.

*Y* = *W*·*X* ≈ *S* (2)

Such a model has been used to capture the essential structure of the data in many applications, including signal separation, feature extraction, and target detection; by taking in consideration some factors, such as the statistical independence between sources, equality between the numbers of mixtures and sources. This requirement however, can be moderated as in [24]. Furthermore, no external noise and such requirement can be resolved during denoising preprocessing. To reduce computational complexity, the data ideally should be centered and whitened. Finally, the non-Gaussianity distribution of the source signals.

Different algorithms are used to measure independence, these output slightly different unmixing matrices. These algorithms can be divided into two main families [25], those based on the minimization of mutual information, and those seeking to maximize the non-Gaussianity of sources.

Mutual information is based on the entropy of random variable, defined by Shannon 1948 to measure of uncertainty. Indeed, there is information about behavior of given system if its entropy value is low. Mutual information can be seen as the reduction of uncertainty regarding a given variable after the observation of another variable. These algorithms seek to minimize mutual information, searching for maximally independent components. We find examples in the literature, especially in [26,27,28,29].

Since ICA assumes that the distribution of each source is not normal or Gaussian, then we can use the non-Gaussianity criterion; as an extracted component is forced to be far as possible from the normal distribution [30].The negentropy measurement can be used to estimate non-Gaussianity by measuring the distance from normality; however, it is difficult to be computed. Hyvärinen and Oja proposed in [31] an approximate formula, that gives birth to the algorithm known as FastICA [32] .

As a statistical technique, FastICA approach results rely on the initialization conditions, the parameterizations of the algorithm and the sampling of the dataset [33]. Therefore, the result of FastICA algorithm should be treated carefully and reliability analysis of the estimated components should be taken in consideration. In this regards, an approach for the estimation of the algorithmic and statistical reliability of the independent components resulting from FastICA called ICASSO, was proposed in [34]. However, ICASSO is an exploratory visualization method that requires users to set the initial parameters and visually interpret the relations between estimates. Furthermore, data dimensionality reduction using PCA is also recommended as a preprocessing step before running the algorithm [34].

In our approach, ICA is used as dimensionality reduction technique after performing DCT to overcome the limitations that compromise the use of ICA in hyperspectral processing.

## 3. DCT

The Discrete Cosine Transform (DCT) represents a sequence of values in terms of a sum of cosine functions oscillating at different frequencies. DCT is similar to the Discrete Fourier Transform (DFT) but uses only real numbers. The complete set of DCTs and Discrete Sine Transforms (DSTs), referred to as discrete trigonometric transforms, was described by Wang and Hunt [35]. DCT concentrates the energy of a signal into a small number of low-frequency DCT coefficients [17].

DCT is employed in many science and engineering applications, such as data lossy compression (e.g., MP3 and JPEG) and spectral methods to solve partial differential equations, numerically.

Assuming that each pixel *x* is a discrete signal represented by a vector *x* = [*x*_0_, *x*_1_, *x*_2_, …, *x_P_*_−1_] in *P* dimensional spectral bands space (*P* is the count of spectral bands), the DCT coefficients of vector *x* are given by [36]:

$$d={\left[d_{0},\;d_{1},\ldots ,d_{P-1}\right]}^{T}$$

(3) $$\{\begin{array}{c} d_{0}=\frac{\sqrt{2}}{P}\sum_{n=0}^{P-1}x_{n}\\ d_{u}=\frac{2}{P}\sum_{n=0}^{P-1}x_{n}\cos\frac{\left(2n+1\right)u\pi }{2P}\;;u=1,2,\ldots ,P-1 \end{array}$$

where *u* is the discrete frequency variable, du is the *u*th DCT coefficient in *P* space dimension and each vector *d* corresponds to the original pixel *x*. Each component of DCT coefficients (DCT coefficients curve) represents the amplitude of a specific cosine base function, proportional to the importance of a cosinusoid present in the pixel spectral curve.

**Figure 1:**
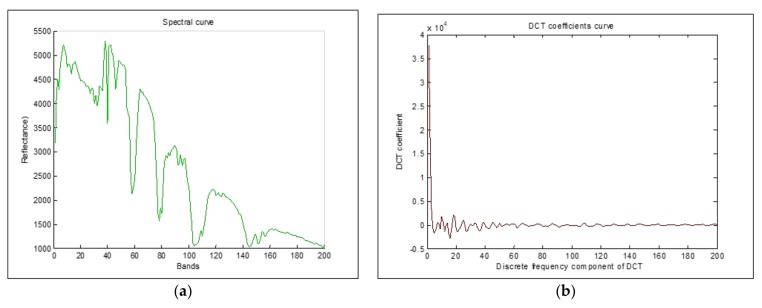
(**a**) Spectral curve, (**b**) DCT Coefficients curve.

An examination of DCT coefficients distribution in Figure 1b, shows that most of the large amplitudes are concentrated in minority of low-frequency components, given the ability of DCT transform to concentrate information energy in a few low frequencies.

In our procedure, we transform the spectral data from the original feature space to a reduced feature space using DCT. We generate a frequency domain profile for the spectral curve of each pixel as illustrated in Figure 1.

**Figure 2:**
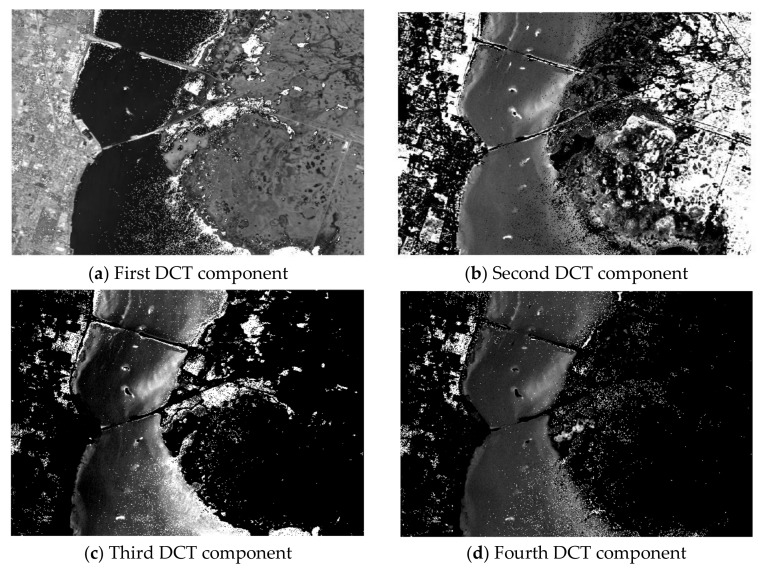
The first four transformed components of the spectral-DCT feature space from the Kennedy Space Center dataset.

**Figure 3:**
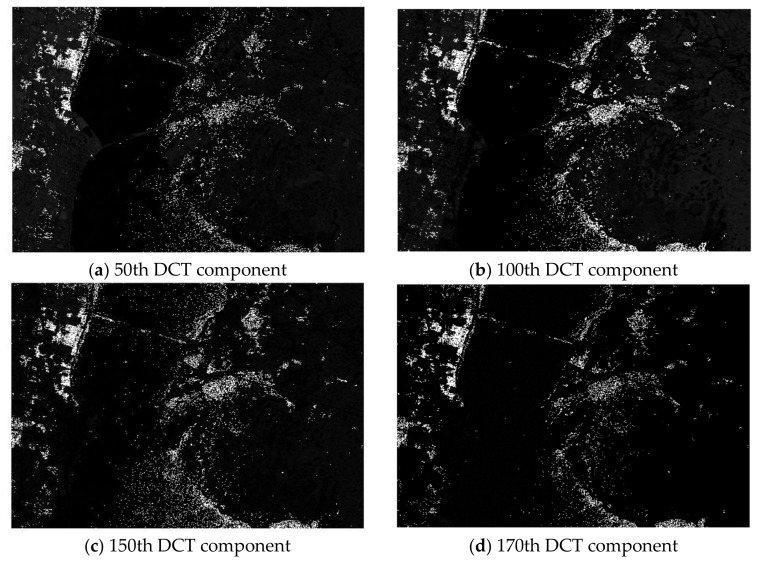
Four transformed high-frequency components of the spectral-DCT feature space from the Kennedy Space Center dataset.

Figure 2 shows the plot of the first four transformed components of the new feature space after performing spectral-DCT on the Kennedy Space Center (KSC) dataset cube. Indeed, we can visually confirm the ability of DCT to concentrate most of the spectral energy in the first low-frequency coefficients. In contrast, Figure 3 presents the plot of four high-frequency components after performing DCT on the same dataset cube (component 50, component 100, component 150, and component 170). For these high-frequency components, the presence of heavy noise is visible, as there is no effective discrimination between the different classes.

## 4. The Proposed Preprocessing Procedure Description 

**Figure 4:**
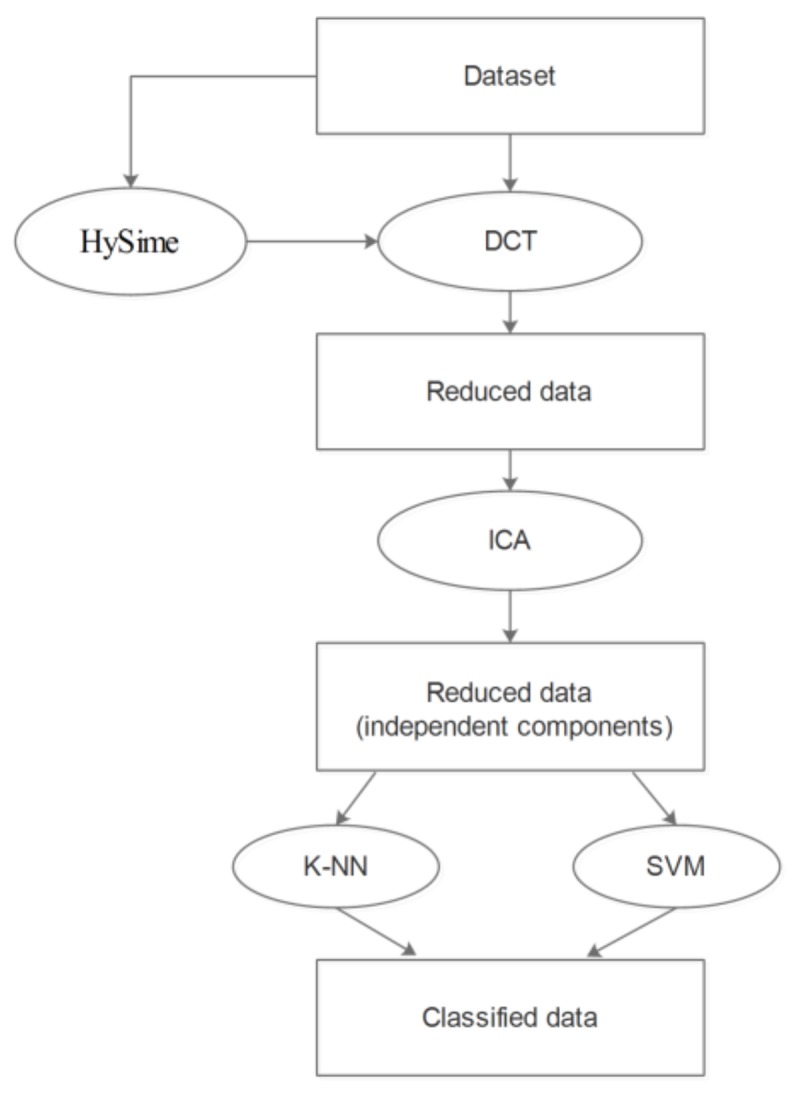
Flowchart of DCT-based preprocessing procedure for ICA.

Our approach is based on applying DCT as preprocessing procedure for ICA to reduce data dimensionality and evaluate its effectiveness by performing classification on the resulting reduced data. The flowchart of our approach is detailed in the following figure (Figure 4).

In this approach we assume that the hyperspectral data cube is a matrix *X_N_*_x*P*_ (*N*: number of pixels, *P*: number of spectral bands) with each row representing a pixel as a 1-D discrete signal *x* = [*x*_0_, *x*_1_, *x*_2_, …, *x_P_*_−1_] in P-dimensional spectral bands space. Applying this formula, the DCT of X is given by *D_N_*_x*P*_ where each row *d* = [*d*_0_, *d*_1_, *d*_2_, …, *d_P_*_−1_] is the DCT coefficients curve of the corresponding vector *x*.

Due to ability of DCT to concentrate energy in a few first coefficients, it is sufficient to take the first *L* (where *L* << *P*) coefficients to preserve the major part of useful information. By taking the first coefficients and discarding the high frequencies that generally represent noise, the result can be considered as denoised data.

The required DCT coefficient number *L* is estimated using Hyperspectral Signal Subspace Identification by the Minimum Error (HySime) method as proposed in [37] to find independent bands in the image for estimating the count of distinct spectral signatures in hyperspectral image. reference [38], demonstrated that by using four real datasets, this method, yields a more accurate estimation than other methods such as Noise-Whitened HFC (NWHFC) and the Harsanyi-Farrand-Chang (HFC) approaches.

After applying DCT and estimating the count of coefficients to be retained using the HySime method, we can now perform ICA on the preprocessed data. To evaluate the effectiveness of our DCT-based preprocessing procedure on ICA, SVM and K-NN classifiers were applied on the present data.

## 5. Data and Evaluation Process

In this section, we present data and our experimental results. We overview the characteristics of the data used in the experimental setup, explain the evaluation process, and the accuracy measurements. Experiments were carried out using Matlab R2014a on a Dual-Core 2 GHz CPU with 8 GB of memory. Specifically, the implementation of the ICA technique was based on the Kurtosis Maximization method.

### 5.1. Data

Two real world remote sensing hyperspectral datasets with different spatial resolution were used for the experiments:

#### 5.1.1. The Indian Pines Dataset

The Indian Pines scene capturing the agricultural Indian Pine area in Northwestern Indiana, USA was recorded by Airborne Visible Infrared Imaging Spectrometer (AVIRIS) at a 3.7 m spatial resolution. This image contains 220 bands and 145 × 145 pixels. Channels affected by noise and water absorption were removed leaving 200 channels. There are 16 classes in the ground reference data. Table 1 describes the classes and gives the number of samples, the number of training and the test points in Indian Pines dataset corresponding to Five-Fold cross-validation model.

**Figure 5:**
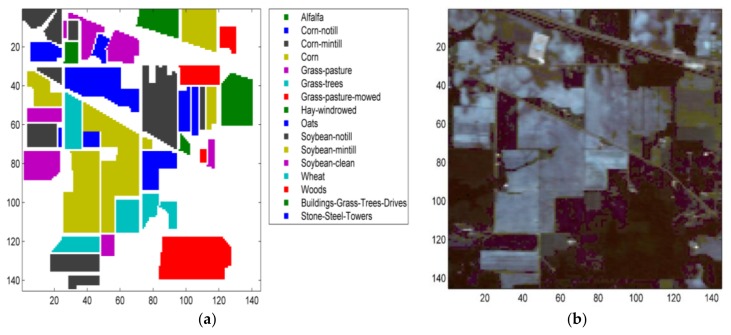
(**a**) Ground reference map of Indian Pines dataset. (**b**) False color composition of the Indian Pines dataset.

Figure 5 shows a ground reference map and a false color composition of the Indian Pines scene.

#### 5.1.2. The Kennedy Space Center (KSC) Dataset

The Kennedy Space Center (KSC), located in Florida, scene was acquired by AVIRIS. This image is 512 × 614 pixels with 18 m spatial resolution. After removing affect water absorption and noisy bands, only 176 bands were retained in our experiments. The ground reference data contains 13 classes. Table 2 describes the classes and gives the number of samples, the number of training and the test points in Kennedy Space Center (KSC) dataset corresponding to Five-Fold cross-validation model.

**Figure 6:**
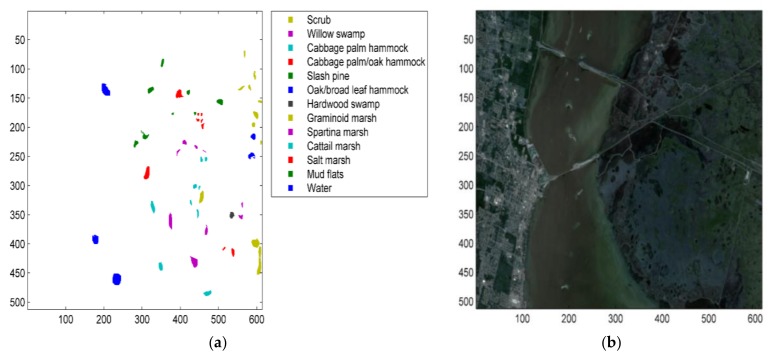
(**a**) Ground reference map of Kennedy Space Center dataset. (**b**) False color composition of Kennedy Space Center dataset.

Figure 6 shows a ground reference map and a false color composition of the Kennedy Space Center scene.

### 5.2. Evaluation Process

To assess the new DCT-based preprocessing procedure for ICA, linear SVM and K-NN classifiers were applied on the experimental datasets after ICA without preprocessing; after ICA with PCA as preprocessing; and after ICA with DCT as preprocessing. In this study, accuracy was estimated using three traditional accuracy measurements, kappa, Average Accuracy (AA), and Overall Accuracy (OA) defined as in [39]:

(4) $$OA=\frac{No.\;of\;pixels\;correctly\;classified}{Total\;no.\;of\;pixels}\times 100$$

(5) $$AA=\frac{\sum_{c=1}^{n}CA_{c}}{n}\times 100$$

where, *CA_c_* is the class wise accuracy of *c*th class, and *n* is the total number of classes in the hyperspectral image. CA is defined by [39]:

(6) $$CA=\frac{No.\;of\;pixels\;correctly\;classified\;in\;each\;class}{Total\;no.\;of\;pixels\;in\;each\;class}\times 100$$

(7) $$Kappa\;coefficients\;=\frac{P\times C-S}{P^{2}-S}$$

where, *P* is the total number of pixels, *C* represents the number of pixels that are accurately classified, and *S* is the sum of the product of rows and columns of the confusion matrix.

The 5-fold cross-validation process was used as the validation model for classification.

## 6. Results and Discussion

### 6.1. Intrinsic Dimension Criterion

In this section, we apply the Hysime criterion for intrinsic dimension estimation to the experimental datasets regarding the three dimensionality reduction techniques: ICA, PCA and DCT. The results are summarized in Table 3.

The results obtained in the following section are based on a selection of 18 components in the Indian pines dataset and 32 components in Kennedy Space Center dataset for ICA, PCA and DCT techniques as listed in Table 3.

### 6.2. ClassificationofIndian Pines and Kennedy Space Centerdatasets 

Table 4 and Table 5 present the classification accuracy of individual class, OA, AA, kappa coefficient, and execution time for the two experimental datasets: Indian Pines and Kennedy Space Center respectively.

A comparison of the accuracy results presented in Table 4 and Table 5, shows that the proposed DCT-ICA method yields the best results than the other tested approaches on almost all of classes. In addition, OA, AA, and kappa coefficient are also larger than those of ICA and PCA-ICA. Specifically, DCT-ICA delivered about (10% to 15%) and (13% to 15%) gain in OA with SVM classifier for Indian Pines and Kennedy Space Center datasets, respectively.

The proposed DCT-ICA approach offers a great improvement in performance, even for classes with few labeled training samples such as class 1, class 7, and class 9, in the Indian Pines dataset; these classes are usually discarded to improve the average classification accuracy [40,41,42,43].

Furthermore, classification execution times (on a Dual-Core 2 GHz CPU with 8 GB of memory) were considerably reduced for the two datasets, using both classifiers. For instance, in Kennedy Space Center dataset, the K-NN execution time was around 0.95 s when performing ICA, around 0.49 s when performing PCA-ICA, and was reduced to around 0.20 s when using our proposed DCT-ICA approach.

**Figure 7:**
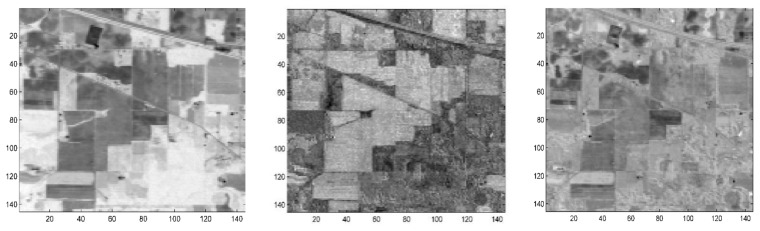
Plot of three arbitrary selected ICs components from the DCT-ICA feature space in the Indian Pines dataset.

**Figure 8:**
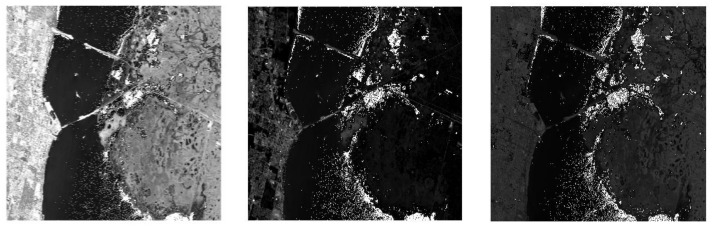
Plot of three arbitrary selected ICs components from the DCT-ICA feature space in the Kennedy Space Center dataset.

Figure 7 and Figure 8 show three arbitrary selected independent components, after performing DCT-ICA, on both the Indian Pines and Kennedy Space Center datasets.

As can be seen in Figure 7 and Figure 8, there is a high contrast between objects, with effective discrimination between the different classes, in almost all the selected independent components after performing DCT-ICA.

Experimental results confirm the superiority of the proposed DCT preprocessing procedure for ICA dimensionality reduction, over all datasets and with both classifiers. We observed significant advantages when we used the DCT preprocessing procedure as classification accuracy was enhanced, overall. In the experiment with the SVM classifier, accuracy improvements using the DCT preprocessing procedure were the most evident. Furthermore, our proposed preprocessing procedure yielded the sharpest improvements in execution time with the K-NN classifier.

## 7. Conclusions

In this study, a novel DCT preprocessing procedure for ICA in hyperspectral dimensionality reduction is proposed. This procedure is based on applying DCT on each pixel spectral curve and estimating the retained coefficients with the HySime method to construct a new reduced feature space where the most useful information is packed in the first low-frequency components. Performing ICA on this reduced feature space overcomes the time consumption problem as well as the absence of specific criteria to select components produced by the ICA. Indeed, useful information is already selected by DCT.

SVM and K-NN classifiers were applied on the reduced data to assess the effectiveness of our approach. Experimental results on two real datasets demonstrate that the proposed preprocessing procedure, DCT-ICA, outperforms ICA without preprocessing and ICA with PCA as a preprocessing technique in terms of accuracy, even for small training sets and short execution times. Indeed, the overall classification accuracy of ICA in the reduced feature space improved by about (10% to 15%), and (13% to 15%) for two experimental datasets, with reduced execution times.

## Figures and Tables

**Table 1:** Classes and number of test and training samples for Indian Pines (corresponding to Five-Fold cross-validation model).

Class	Type	Samples	Training	Testing
1	Alfalfa	46	36	10
2	Corn-notill	1428	1142	286
3	Corn-mintill	830	664	166
4	Corn	237	189	48
5	Grass-pasture	483	386	97
6	Grass-trees	730	584	146
7	Grass-pasture-mowed	28	22	6
8	Hay-windrowed	478	382	96
9	Oats	20	16	4
10	Soybean-notill	972	777	195
11	Soybean-mintill	2455	1964	491
12	Soybean-clean	593	474	119
13	Wheat	205	164	41
14	Woods	1265	1012	253
15	Buildings-Grass-Trees-Drives	386	308	78
16	Stone-Steel-Towers	93	74	19

**Table 2:** Classes and number of test and training samples for Kennedy Space Center (KSC) dataset (corresponding to Five-Fold cross-validation model).

Class	Type	Samples	Training	Testing
1	Scrub	875	700	175
2	Willow swamp	279	223	56
3	Cabbage palm hammock	294	235	59
4	Cabbage palm/oak hammock	290	232	58
5	Slash pine	185	148	37
6	Oak/broad leaf hammock	263	210	53
7	Hardwood swamp	121	96	25
8	Graminoid marsh	496	396	100
9	Spartina marsh	598	478	120
10	Cattail marsh	465	372	93
11	Salt marsh	482	385	97
12	Mud flats	578	462	116
13	Water	1066	852	214

**Table 3:** Intrinsic dimension estimation.

Criterion	Indian Pines	KSC
**Hysime**	18	32

**Table 4:** Classification accuracy (%) using K-NN and SVM classifiers on the Indian Pines Dataset.

Classes	K-NN			SVM		
	ICA	DCT-ICA	PCA-ICA	ICA	DCT-ICA	PCA-ICA
1	58.89	**78.44**	76.00	80.22	**86.67**	72.00
2	66.45	**74.93**	69.54	61.84	**82.71**	61.63
3	47.35	**65.18**	63.01	25.18	**64.46**	44.58
4	46.84	**50.59**	50.25	42.65	**78.42**	52.33
5	88.20	**94.20**	90.46	91.09	93.17	**93.37**
6	96.99	**97.81**	97.40	94.79	96.03	**96.30**
7	76.00	**83.33**	82.67	69.33	**92.67**	82.67
8	98.53	**98.95**	98.13	98.11	**99.58**	97.48
9	35.00	**60.00**	35.00	25.00	**90.00**	65.00
10	69.13	**80.45**	76.54	30.66	**69.55**	53.60
11	73.32	**81.47**	77.52	75.40	**75.15**	74.01
12	39.12	**59.86**	50.77	9.27	**72.35**	26.63
13	93.66	**99.02**	95.61	95.12	**96.59**	93.66
14	93.60	**94.86**	93.91	96.28	**96.52**	95.81
15	43.52	**49.21**	44.32	54.16	**66.54**	54.66
16	92.40	**94.56**	93.39	94.56	**97.84**	95.67
**Kappa (%)**	68.74	**77.86%**	73.90	60.50	**78.61**	66.47
**OA (%)**	72.66	**80.61%**	77.15	66.06	**81.28**	70.87
**AA (%)**	69.94	**78.93%**	74.66	65.23	**84.89**	72.46
**Time (s)**	4.1755	**0.44037**	0.77588	140.8222	**93.5459**	128.1354

Bolded values denote the best results.

**Table 5:** Classification accuracy using K-NN and SVM classifiers on the KSC Dataset.

Classes	K-NN			SVM		
	ICA	DCT-ICA	PCA-ICA	ICA	DCT-ICA	PCA-ICA
1	89.23	**94.22**	91.59	92.12	**96.58**	91.59
2	74.49	**87.24**	82.30	78.62	**97.53**	76.99
3	59.75	**91.00**	77.35	78.88	**92.16**	80.46
4	37.32	**66.26**	41.35	53.15	**87.72**	44.39
5	47.90	**55.34**	49.20	52.18	**82.61**	47.23
6	22.26	**45.45**	27.93	48.93	**84.27**	43.59
7	62.86	**88.57**	56.19	64.76	**93.33**	63.81
8	71.00	**87.00**	71.25	76.81	**95.37**	61.46
9	86.15	**95.00**	86.92	90.19	**98.85**	86.54
10	48.74	**92.81**	69.04	77.74	**98.52**	78.96
11	95.23	**97.38**	95.46	96.18	**99.52**	97.85
12	63.63	**90.05**	70.20	75.77	**96.81**	79.72
13	98.92	**99.35**	99.35	99.57	**99.89**	99.68
**Kappa (%)**	71.63	**87.80**	76.47	80.81	**95.62**	78.68
**OA (%)**	74.61	**89.06**	78.93	82.77	**96.07**	80.86
**AA (%)**	65.96	**83.82**	70.63	75.76	**94.09**	73.25
**Time (s)**	0.9565	**0.2033**	0.4924	60.962	**9.8957**	53.9262

Bolded values denote the best results.
